# Organoruthenium(II) complexes of acetazolamide potently inhibit human carbonic anhydrase isoforms I, II, IX and XII

**DOI:** 10.1080/14756366.2018.1547288

**Published:** 2019-01-03

**Authors:** Sara Seršen, Katja Traven, Jakob Kljun, Iztok Turel, Claudiu T. Supuran

**Affiliations:** a Faculty of Chemistry and Chemical Technology, University of Ljubljana, Ljubljana, Slovenia;; b Dipartimento Neurofarba, Sezione di Scienze Farmaceutiche e Nutraceutiche, Università degli Studi di Firenze, Florence, Italy

**Keywords:** Carbonic anhydrase, human isoforms, acetazolamide, Ru(II), metal complex

## Abstract

Two acetazolamide (AAZ) complexes with ruthenium(II) η^6^-*p*-cymene chloride were synthesised, characterised and tested for their inhibitory effects on several carbonic anhydrase (CA, EC 4.2.1.1) isoforms with pharmacological applications. Against human (h) isoform hCA I, the two complexes showed inhibition constants in the range of 8.5–23.4 nM (AAZ has a K_I_ of 250 nM), against hCA II of 0.48–4.2 nM, whereas against hCA IX of 0.63–3.8 nM and against hCA XII of 0.04–0.52 nM, respectively. These highly effective ruthenium acetazolamide derivatives against the tumour-associated CA isoforms IX and XII warrant further *in vivo* studies, in hypoxic tumours overexpressing these enzymes.

## 1. Introduction

**Figure 1.**
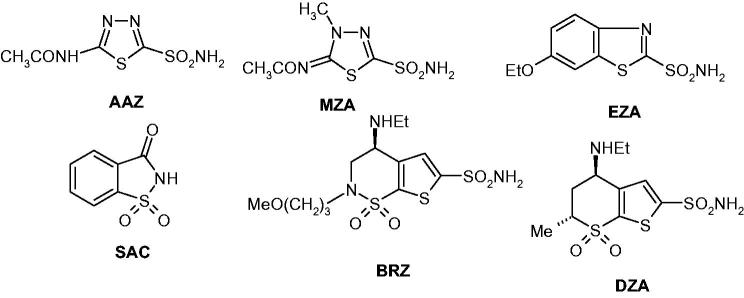
Clinically used sulfonamides with CA inhibitory activity^1–3^.

The carbonic anhydrases (CAs, EC 4.2.1.1) are a superfamily of metalloenzymes acting as efficient catalysts for the hydration of CO_2_ to bicarbonate and protons, as well as for the opposite reaction, the “dehydration” of bicarbonate in the presence of hydronium ions, leading to CO_2_^1–3^. Since this equilibrium is involved in pH/CO_2_ homeostasis, respiration, secretion of electrolytes and several metabolic pathways, among which lipogenesis, such as urea biosynthesis, gluconeogenesis, etc., the activity of these enzymes is correlated with many physiological and pathological conditions, making them attractive drug targets for a variety of disorders^3–9^. In fact, CAs are present not only in humans (with 15 different isoforms described to date) but they are also widespread in organisms all over the phylogenetic tree, with seven distinct genetic families reported, the α-, β-, γ-, δ-, η-, ζ- and θ-CAs^1^^,^^2^. CA inhibitors (CAIs) belonging to the sulfonamide (and sulfamate) types are clinically used for several decades as diuretics^4^, antiglaucoma agents^5^, antiobesity drugs^6^, and more recently, a number of studies showed that CA inhibition has profound antitumour effects by inhibition of hypoxia-inducible isoforms CA IX and XII, overexpressed in many hypoxic tumours^7^. Furthermore, several proof-of-concept studies demonstrated the involvement of some CA isoforms in neuropathic pain^8^ and arthritis^9^, with the CAIs of sulfonamide/coumarin^10^ types demonstrating significant effects *in vivo*, in animal models of these diseases. Thus, the field of drug design, synthesis and *in vivo* investigations of various types of CAIs is a highly dynamic one, with a large number of interesting new chemotypes acting on these widespread enzymes constantly emerging^9^^,^^10^. Among the clinically used sulfonamide CAIs are acetazolamide (**AAZ**), methazolamide (**MZA**), ethoxzolamide (**EZA**), saccharin (**SAC**), brinzolamide (**BRZ**) and dorzolamide (**DRZ**) – Figure 1^1–3^.

The sulfonamides are highly effective CAIs, with the heterocyclic and some aromatic compounds acting in the low nanomolar range^1–3^, but their main drawback is that they are generally non-selective for the many CA isoforms which must be targeted for various pharmacological applications^4–9^. Thus, alternative chemotypes for a diverse approach to CA inhibition were developed^1–7^. One of them was that of obtaining coordination compounds in which the sulfonamides act as ligands to various transition or main group metal ions, leading to sulfonamide metal complexes^11^. Originally investigated for obtaining transition metal ion complexes of acetazolamide **AAZ**, methazolamide **MZA** and ethoxzolamide **EZA** (the main sulfonamide, clinically used drugs belonging to this class of pharmacological agents)^11^, this approach was subsequently extended to a large set of primary and secondary aromatic/heterocyclic sulfonamides, also including the clinical drugs saccharin (**SAC**), brinzolamide (**BRZ**) and dorzolamide (**DRZ**)^12–17^. Other sulfonamides possessing a diverse scaffold but effective CA inhibitory properties were also included in such studies together with metal ions which may add a supplementary pharmacological activity, such as Pt(II), Pd(II) and Ru(II) for the antitumour effects^11^^,^^17^^,^^18^, zinc(II) for the antiglaucoma action^14^, Al(III) for antacid properties^13^, Co(II), Ag(I) and Cu(II) for antifungal activity^13^. Imaging tumours overexpressing some CA isoforms (e.g., CA IX and XII) with sulfonamide complexes incorporating isotopes of metal ions which emit positrons (for PET imaging), such as Ga(III), In(III) or Cu(II) were also investigated^17^, allowing interesting developments in the field.

Considering the interest of one of our groups for the investigation of Ru(II) complexes with various ligands resulting in versatile types of biological activities (antitumour, antibacterial, enzyme inhibitory, etc.)^19^^,^^20^, we report in this communication the preparation and investigation as CA inhibitors of organoruthenium(II) complexes of acetazolamide, which were found to possess subnanomolar affinity for many pharmacologically relevant isoforms, such as the cytosolic CA I and II and the transmembrane, tumour-associated CA IX and XII.

## 2. Materials and methods

### 2.1. Chemistry

The two acetazolamide organoruthenium acetazolamide complexes **1** and **2** were prepared as reported in Biancalana et al.^18^ with small modifications described below. H NMR spectra were recorded on a Bruker Avance III 500 spectrometer (at room temperature and 500.10 MHz) using TMS as an internal standard in (CD_3_)_2_CO. Infrared spectra were recorded with a PerkinElmer Spectrum 100 FTIR spectrometer, equipped with a Specac Golden Gate Diamond ATR as a solid sample support. All NMR data processing was carried out using MestReNova, version 8.1.2.

Complex **1**: [(η6-p-cymene)RuCl(μ-Cl)]_2_ (240 mg, 0.780 mmol) and acetazolamide **AAZ** (180 mg, 0.810 mmol, 1.03 eq.) were dissolved in 30 mL of a acetone solution for 12 h at room temperature. The solvent was removed and 5 mL of CHCl_3_ was added. The product **1** was filtered off and left to dry at 55 °C for 4 h. Yield: 47% (277 mg), red solid. ^1^H NMR (Acetone-d_6_, 500 MHz): δ = 11.97 (s, 1H, AAZ C(N*H*)C), 7.81 (s, 2H, –SO_2_N*H*_2_), 5.74 (d, 2H, J = 5.8 Hz, Ar–*H* cymene), 5.48 (d, 2H, J = 6.0 Hz, Ar–*H* cymene), 3.08 (hept, 1H, J = 7.0 Hz, cymene Ar–C*H*(CH_3_)_2_), 2.25 (s, 3H, AAZ Ar–C*H*_3_), 2.18 (s, 3H, cymene C*H*_3_), 1.27 (d, 3H, J = 7.0 Hz, cymene Ar–CH(C*H*_3_)_2_) ppm. IR selected bands (cm^−1^, ATR): 3146 (νN–H), 3060 (νC = C), 1709 (νC = O), 1521 (νC = N), 1368, 1353, 1311 (ν_as_SO_2_), 1173 (ν_s_SO_2_), 683, 620.

Complex **2**: Complex **1** (200 mg, 0.38 mmol) was added to chromatography column with silica gel as stationary phase and acetone as mobile phase. The solvent was removed and 2 mL of CH_2_Cl_2_ and 30 mL of heptane was added to precipitate the product. The product was filtered off and left to dry at 55 °C for 4 h. Rf factor of complex **2** was found to be between 0.45 and 0.55. Yield: 60% (120 mg), yellow solid. ^1^H NMR (Acetone-d_6_, 500 MHz): *δ* = 12.02 (br, 1H, AAZ C(N*H*)C), 5.66 (m, 2H, Ar–*H* cymene), 5.43 (m, 2H, Ar–*H* cymene), 3.00 (s, 1H, –SO_2_N*H*), 2.96, (hept, 1H, J = 6.7 Hz, cymene Ar–C*H*(CH_3_)_2_), 2.38 (s, 3H, AAZ C*H*_3_), 2.16 (s, 3H, cymene Ar–C*H*_3_), 1.31 (d, J = 7.0 Hz, 6H, cymene Ar–CH(C*H*_3_)_2_). IR selected bands (cm^−1^, ATR): 3170 (νN–H), 2965 (νC–C), 1701 (νC = O), 1523 (νC = N), 1372, 1310 (ν_as_SO_2_), 1289, 1155 (ν_s_SO_2_), 920, 633.

### 2.2. CA enzyme inhibition assay

An Sx.18Mv-R Applied Photophysics (Oxford, UK) stopped-flow instrument has been used to assay the catalytic activity of various CA isozymes for CO_2_ hydration reaction^21^. Phenol red (at a concentration of 0.2 mM) was used as indicator, working at the absorbance maximum of 557 nm, with 10 mM Hepes (pH: 7.5, for α-CAs) or TRIS (pH: 8.3, for β- and γ-CAs) as buffers, 0.1 M Na_2_SO_4_ (for maintaining constant ionic strength), following the CA-catalysed CO_2_ hydration reaction for a period of 10 s at 25 °C. The CO_2_ concentrations ranged from 1.7 to 17 mM for the determination of the kinetic parameters and inhibition constants. For each inhibitor, at least six traces of the initial 5%–10% of the reaction have been used for determining the initial velocity. The uncatalysed rates were determined in the same manner and subtracted from the total observed rates. Stock solutions of inhibitors (10 mM) were prepared in distilled-deionised water and dilutions up to 1 nM were done thereafter with the assay buffer. Enzyme and inhibitor solutions were pre-incubated together for 15 min (standard assay at room temperature) prior to assay, to allow for the formation of the enzyme–inhibitor complex. The inhibition constants were obtained by non-linear least-squares methods using PRISM 3 and the Cheng–Prusoff equation, as reported earlier^22^. All CAs were recombinant proteins produced as reported earlier by our group^23^^,^^24^.

## 3. Discussion and conclusion

Two of the organoruthenium AAZ complexes were described recently by Biancalana et al.^18^ and were also isolated and characterised in this work. Herein, we would just like to mention some differences between our procedure and the one reported in the literature^18^. Complex **1** was prepared very similarly in both laboratories, from the sulfonamide ligand and [(η^6^-*p*-cymene)RuCl(μ-Cl)]_2_. We have performed the reaction for 12 h at room temperature (yield 47%), whereas Biancalana et al.^18^ performed the reaction at room temperature for 3 days obtaining 70% yield. By refluxing 7 h, the yield was much higher (90%). The preparation of complex **2** was, however, much different between these two laboratories. Biancalana et al.^18^ isolated product **2** via addition of base (NaOH) to the starting reaction mixture, or by previously preparing the acetazolamide sodium salt which was subsequently reacted with the organoruthenium derivative. In our case, a solution of complex **1** in acetone was added to a chromatography column filled with silica gel as stationary phase. The solvent was removed and addition of a mixture of dichloromethane and heptane led to the precipitation of product **2**. We hypothesise that in our procedure, the acid–base reaction leading to the deprotonation of acetazolamide occurred in the column, being probably assisted by the chromatographic stationary phase. As far as we know, this is the first report in which a deprotonated acetazolamide complex is obtained without the use of a strong base such as sodium/potassium hydroxide or tertiary amines.

**Figure 2.**
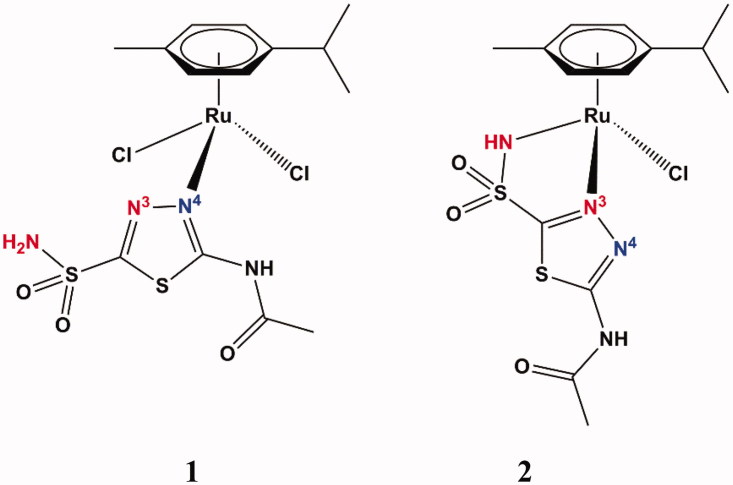
Structure of acetazolamide organoruthenium complexes **1** (left) and **2** (right). The X-ray crystallography for these compounds was reported in Biancalana et al.^18^

The structures of the two complexes **1** and **2** are shown in Figure 2, and they were obtained by X-ray crystallography in both laboratories but reported first by Biancalana et al.^18^

In complex **1**, the sulfamoyl moiety of **AAZ** is not involved in the interaction with the metal ion, which apart the *p*-cymene ligand has two chloride anions and the endocyclic N4 (Figure 2 – blue) from the 1,3,4-thiadiazole ring as ligands, in the pseudo-tetrahedral geometry characteristic of Ru(II) – Figure 2. The second complex (**2**) is also pseudo-tetrahedral, with the Ru(II) ion being complexed to the cymene ring, one chloride anion and acetazolamide acting as bidentate ligand, with the deprotonated sulfonamide nitrogen atom and the endocyclic N3 (Figure 2 – red) interacting with the metal ion (Figure 2). Thus, as already documented earlier^11–16^^,^^18^, this heterocyclic sulfonamide may act as a very versatile ligand when interacting with metal ions for the formation of complexes.

Data of Table 1 show that the two acetazolamide complexes are much more effective as inhibitors of four physiologically relevant isoforms, the cytosolic CA I and II and the transmembrane, tumour-associated CA IX and XII, involved in several pathologies such as glaucoma (CA II and XII)^3^^,^^5^, or tumours^7^ and arthritis^9^ (CA IX and XII) as **AAZ** itself. Both complexes were several fold better CAIs compared to the parent ligand, but the complex **2**, in which the sulfonamide moiety was deprotonated, acted as much better inhibitor compared to **1**. It should be mentioned that **AAZ** itself is a highly effective, clinically used inhibitor (Table 1).

**Table 1.** Inhibition of human (h) CA isoforms hCA I, II, IX and XII with acetazolamide (**AAZ**) and its two Ru(II) derivatives **1** and **2**, by a stopped flow, CO_2_ hydrase assay.

	K_I_ (nM)*			
Compound	hCA I	hCA II	hCA IX	hCA XII
**AAZ**	250	12.0	25.2	5.7
**1**	23.4	4.2	3.8	0.52
**2**	8.5	0.48	0.63	0.04

* Mean from three different assays. Errors were in the range of ±5%–10% of the reported values (data not shown).

Against hCA I, the two complexes showed inhibition constants in the range of 8.5–23.4 nM (**AAZ** has a K_I_ of 250 nM), against hCA II of 0.48–4.2 nM, whereas against hCA IX of 0.63–3.8 nM and against hCA XII of 0.04–0.52 nM.

Although these two complexes have such a high affinity for the enzyme, we were unable to determine the X-ray crystal structure of a CA isoform (such as CA II or I) in adduct with one of them, due to the fact that no good quality crystals were possible to obtain in various crystallisation conditions that we used. Thus, the inhibition mechanism with these two acetazolamide derivatives remains for the moment unknown. Biancalana et al.^18^ have also discovered that **1** is involved in various equilibria in solution and one of the products is also **2**. However, as seen from Table 1, stronger (bidentate) binding of ligand, obviously, results in higher activity.

Although Biancalana et al.^18^ reported that compounds **1** and **2** were not cytotoxic to some cancer cell lines as well as to non-tumorigenic cells, we think that by considering the results of Can et al.^17^^a^ and Dilworth et al.^17^^b^ it is worth testing the antitumour effects of such derivatives in hypoxic tumour cell lines in which CA IX and XII are present. The negative results reported by Biancalana et al.^18^ are due to the fact that the experiments (in human ovarian and kidney tumour cell lines) were performed in normoxia and not hypoxia. In fact, only in hypoxic conditions, there is a relevant overexpression of CA IX/XII with the consequent potent antitumour effect shown by the CAIs, such as, for example, the sulfonamide compound in Phase II clinical trials SLC-0111^25^.
